# Construction of *Cupriavidus necator* displayed with superoxide dismutases for enhanced growth in bioelectrochemical systems

**DOI:** 10.1186/s40643-023-00655-2

**Published:** 2023-06-28

**Authors:** Ke Chen, Chunling Ma, Xiaolei Cheng, Yuhua Wang, Kun Guo, Ranran Wu, Zhiguang Zhu

**Affiliations:** 1 Key Laboratory of Industrial Fermentation Microbiology, Ministry of Education, Tianjin Key Laboratory of Industrial Microbiology, The College of Biotechnology, Tianjin University of Science and Technology, Tianjin, 300457 China; 2 University of Chinese Academy of Sciences, 19A Yuquan Road, Shijingshan District, Beijing, 100049 China; 3 Haihe Laboratory of Synthetic Biology, 21 Xishiwudao, Tianjin Airport Economic Park, Tianjin, 300308 China; 4 Key Laboratory of Engineering Biology for Low-Carbon Manufacturing, Tianjin Institute of Industrial Biotechnology, Chinese Academy of Sciences, 32 Xiqidao, Tianjin Airport Economic Park, Tianjin, 300308 China; 5 School of Chemical Engineering and Technology, Xi’an Jiaotong University, Xi’an, 710049 China

**Keywords:** Carbon dioxide, Single cell protein, Bioelectrochemical system, Cell surface display, Hydrogen-oxidizing bacteria

## Abstract

It is of great significance to utilize CO_2_ as feedstock to synthesize biobased products, particularly single cell protein (SCP) as the alternative food and feed. Bioelectrochemical system (BES) driven by clean electric energy has been regarded as a promising way for *Cupriavidus necator* to produce SCP from CO_2_ directly. At present, the key problem of culturing *C. necator* in BES is that reactive oxygen species (ROS) generated in cathode chamber are harmful to bacterial growth. Therefore, it is necessary to find a solution to mitigate the negative effect of ROS. In this study, we constructed a number of *C. necator* strains displayed with superoxide dismutase (SOD), which allowed the decomposition of superoxide anion radical. The effects of promoters and signal peptides on the cell surface displayed SOD were analyzed. The proteins displayed on the surface were further verified by the fluorescence experiment. Finally, the growth of *C. necator* CMS incorporating a pBAD-SOD-E-tag-IgAβ plasmid could achieve 4.9 ± 1.0 of OD_600_ by 7 days, equivalent to 1.7 ± 0.3 g/L dry cell weight (DCW), and the production rate was 0.24 ± 0.04 g/L/d DCW, around 2.7-fold increase than the original *C. necator* CMS (1.8 ± 0.3 of OD_600_). This study can provide an effective and novel strategy of cultivating strains for the production of CO_2_-derived SCP or other chemicals in BES.

**Graphical Abstract:**

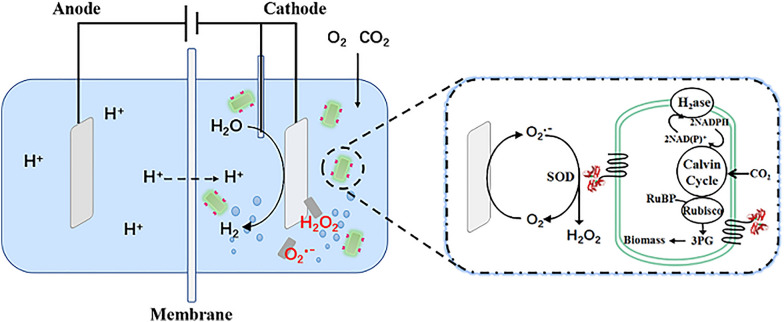

**Supplementary Information:**

The online version contains supplementary material available at 10.1186/s40643-023-00655-2.

## Introduction

The environmental problems and climate change caused by CO_2_ drive the research for a clean and efficient CO_2_ fixation and utilization scenario. With the prosperous development of synthetic biotechnology, scientists have gradually opened up a way to convert CO_2_ to various products by biological tools including engineered microorganisms or designed enzymes (Wang et al. 2020). Different from sugar or biomass-derived feedstocks, biotransformation of CO_2_ faces challenges of inertness of CO_2_ molecule and inadequate reducing power from biosystems themselves, leading to slow reaction rate and low conversion efficiency. Recently, renewable electricity-driven microbial electrosynthesis in bioelectrochemical systems (BES) has been emerged as one of the promising technologies for CO_2_ biotransformation by overcoming thermodynamic and kinetic barriers of the process (Li et al. 2022; Shang et al. 2023). Particularly, via BES, CO_2_-derived microbial single cell protein (SCP) has been proposed as a potential alternative to revolutionize the production mode of food and feed in a carbon neutral or negative way (Mishra et al. 2020; Yang et al. 2022b). This technology has the potential merits of enhancing the production rate, saving land and water usage, and improving overall energy conversion efficiency (Guo et al. 2021; Wong et al. 2014).

Hydrogen-oxidizing bacteria (HOB) that can autotrophically capture CO_2_ have been proposed as a typical choice for CO_2_-derived SCP in BES (Yang et al. 2022a). The metabolic features allow HOB to grow on H_2_ (electron donor) and O_2_ (electron acceptor) while fixing CO_2_ by the Calvin–Benson–Bassham cycle into cell mass and assimilating nitrogen into high quality protein (Lin et al. 2022). Among different types of HOB, *Cupriavidus necator* (formally called *Ralstonia eutropha*) is usually regarded as a model autotrophic organism and could be a promising producer of SCP (Burgdorf et al. 2005; Yu 2018). *C. necator* is a Gram-negative bacterium that populates soil and fresh water habitats at the aerobic-anaerobic interface and belongs to the class of Betaproteobacteria. The gene expression system and genome editing CRISPR-Cas9 technique have already been studied for *C. necator* (Hu et al. 2020; Xiong et al. 2018). The obtained biomass has been found with a high crude protein content (approx. 50–80%), which therefore is referred to as SCP rather than vegetable protein (Ritala et al. 2017; Volova and Barashkov 2010). Given the interesting feature, *C. necator* is already proposed as a possible protein source within biological life support systems for space missions, as well as for human and animal nutrition (Kunasundari et al. 2013; Repaske and Mayer 1976; Yu 2018).

Although with these prosperous potential applications, techniques for growing *C. necator* in BES have not been well developed. One of the most important issues is that reactive oxygen species (ROS), which are generated during the cultivation, are harmful to bacterial growth. For example, Holtmann et al. constructed an engineered *C. necator* to synthesize a high-value terpene, α-humulene, in BES, and ~ 0.8 g/L of dry cell weight (DCW) was achieved, equivalent to the OD_600_ of ~ 2.0 (Krieg et al. 2018). The similar OD_600_ level was demonstrated in another example of using an engineered *C. necator* to synthesize lycopene in BES (Wu et al. 2022). So was the other example of culturing *C. necator* in a hybrid biological-inorganic electrochemical system for producing multi-carbon products (Liu et al. 2018). To tackle the ROS issue, Liao et al. used a porous ceramic cup to cover the electrode and extend the ROS diffusion time so that the negative effect on bacterial growth could be mitigated (Li et al. 2012). Nocera et al. analyzed the types and conditions of ROS and added the catalase (CAT) to the cathodic chamber to decompose ROS and improve the product yield. However, these methods did not work very effectively on the growth of bacteria, e.g., with slightly incremental OD_600_ (Torella et al. 2015). Besides CAT, superoxide dismutase (SOD) which can clear superoxide anion radical (O_2_^−^) may also be a feasible enzyme to reduce the inhibition of ROS in BES. Moreover, it would be a new strategy if the enzymes decomposing ROS could be displayed on the surface of *C. necator*, rather than being added exogenously, to form a shield which can protect the growth of bacteria, since the goal is to produce SCP and adding other protein into the system would not be an ideal solution.

Cell surface display technology is to enrich foreign proteins or peptides on the cell surface by fusing an anchoring protein with the protein or peptide of interest. It has been widely used for various biotechnological applications such as the screening of protein libraries, whole cell biocatalysis, etc. (Nicolay et al. 2015). Many studies have shown that enzymes can be displayed on the cell surface of gram-negative bacteria while maintaining their relative spatial structure and biological activity (Liu et al. 2022; Tozakidis et al. 2014). A number of anchoring proteins have been reported previously to allow for translocating fusion protein to cell outer membrane, including INP (a secretory outer membrane protein) (Fan et al. 2011), Fhua (an outer membrane receptor for ferrichrome-iron) (Etz et al. 2001), OmpA (a truncated outer membrane protein A) (Verhoeven et al. 2009), Lpp-OmpA (a chimeric protein containing the signal sequence of lipoprotein) (Earhart 2000; Lu et al. 2014), and IgAβ (the β domain of immunoglobulin A) (Biondo et al. 2012; Valls et al. 2000). However, in *C. necator*, there is rather limited report pertaining to the cell surface display. In one example, Fernández et al. displayed a heavy metal adsorbing protein, metallothionein I, onto the cell surface of *C. necator* CH34 strain which, therefore, could thrive in soils highly polluted with metal ions (Valls et al. 2000). Another example is an enzyme complex displayed for the biological conversion of CO to CO_2_ (Hyeon et al. 2015)_._ These endeavors inspire us to evaluate more surface displaying conditions with *C. necator* and to construct a strain displayed with ROS decomposing enzymes to improve its growth in BES.

**Scheme 1:**
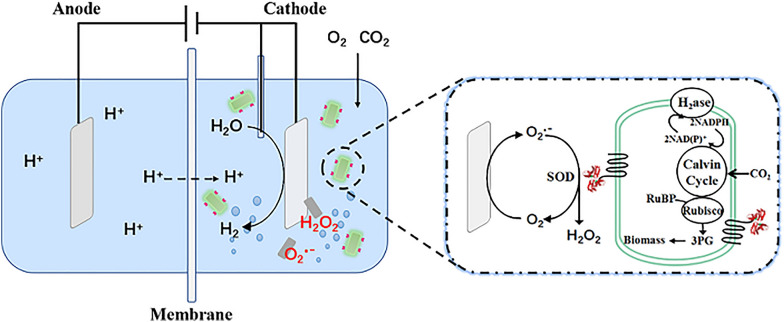
Schematic of the BES for *C. necator* cultivation and SCP production. SOD displayed on the cell surface can consume superoxide anion radical generated from the cathode

In this study, we constructed a number of *C. necator* strains displayed with the SOD enzyme (Scheme 1). The effects of promoters and signal peptides on cell surface display were analyzed. And the protein displayed on the surface was verified by the fluorescence experiment. It was demonstrated that *C. necator* grew better after being displayed with SOD possibly owing to the elimination of the negative effect of O_2_^−^. This study can provide an effective and novel strategy of cultivating strains for the production of CO_2_-derived SCP or other chemicals in BES.

## Material and methods

### Strains and reagents

*E. coli* S17 was used for gene cloning. *C. necator* H16 and CMS were obtained from Professor Changhao Bi’s group (Tianjin Institute of Industrial Biotechnology, Chinese Academy of Sciences). The latter was described by Li et al. (2020) and used as the original strain for all engineered *C. necator*. The primers were synthesized by GENEWIZ (Beijing, China) and listed in Additional file 1: Table S1. PrimeSTAR Max DNA polymerase was purchased from Takara (Dalian, China). All enzymes for molecular biology experiments were purchased from New England Biolabs (Ipswich, MA, U. S.). Catalase was kindly gifted by Auwikey (Suzhou, China). Pyrogallol and L-arabinose were purchased from Sigma (Shanghai, China). All immunofluorescence reagents were from Beyotime (Shanghai, China), except for the rabbit anti-E-tagged polyclonal antibody which was from Thermo Fisher (Shanghai, China).

### *C. necator* strains activation and construction

The strains were activated in LB solid plates with 10 μg/mL gentamycin at 30 °C for 48 h firstly and then can be used to culture. A fusion gene SOD-IgAβ was constructed with the SOD from *Thermus thermophilus* (32 kD) and the IgA protease with β-domain (50 kD) from *Neisseria gonorrhoeae*. The SOD-IgAβ was also tagged with a short peptide epitope (E-tag) for immunodetection with a polyclonal antibody (anti-E-tag-pAb). Based on SOD-E-tag-IgAβ, different promoters were selected. Additionally, plasmids pBAD-SOD and pBAD-pelB-SOD were applied to explore the effects of intracellular overexpression and extracellular secretion of the SOD. For further validation, the SOD was replaced by red fluorescent protein (mCherry) for fluorescence microscopy. The plasmids used in this study are listed in Table 1. Cloning was performed in *E. coli* S17 and proteins were expressed in *C. necator* CMS as detailed in the next section. All plasmids were constructed using the Prolonged Overlap Extension PCR technique (You et al. 2012; You and Zhang 2014).

**Table 1:** Plasmids used in this study

Plasmid	Description
BBa_J23100-SOD-E-tag-IgAβ	pBBR1 derived, SOD-E-tag-IgAβ was drived by BBa_J23100
T7-SOD-E-tag-IgAβ	pBBR1 derived, SOD-E-tag-IgAβ was drived by T7 promoter
pBAD-pelB-SOD-E-tag-IgAβ	pBBR1 derived, pelB-SOD-E-tag-IgAβ was drived by pBAD
pBAD-mCherry	pBBR1 derived, mCherry was drived by pBAD
pBAD-mCherry-E-tag-IgAβ	pBBR1 derived, mCherry-E-tag-IgAβ was drived by pBAD
pBAD-SOD	pBBR1 derived, SOD was drived by pBAD
pBAD-pelB-SOD	pBBR1 derived, pelB-SOD was drived by pBAD

### Protein expression

The *E. coli* Top 10 harboring plasmids encoding the SOD was grown in LB medium with 100 μg/mL ampicillin at 37 °C. For liquid culture, once OD_600_ of the cultured cell reached 0.8–1.0, 0.1 mM IPTG and 2 mM MnCl_2_ were added to induce protein expression at 30 °C for 10 h (Liu et al. 2011). Cell pellets were harvested by centrifugation and then were resuspended in 20 mM 4-(2-hydroxyethyl)-1-piperazineethanesulfonic acid (HEPES) buffer (pH 7.5) containing 50 mM NaCl. After high pressure homogenization and centrifugation, the target protein in the supernatant was heat treated with 70 °C for 20 min. The protein concentration was determined by the Bradford assay using bovine serum albumin (BSA) as the standard.

The activated *C. necator* strains were grown in LB medium with 10 μg/mL gentamycin at 30 °C for 8 h. Cell pellets were harvested by centrifugation and then were resuspended in 0.9% NaCl buffer (pH 7.0). The cells were lysed by ultrasonication (10 min, 3 s, 3 s, 25 °C, 60%). After centrifugation at 8000 g for 15 min, 10 µL of the total protein, supernatant and precipitate were loaded into 12% sodium dodecyl sulfate polyacrylamide gel electrophoresis (SDS-PAGE) to check their expression level.

### Determination of SOD activity by pyrogallol autoxidation

The inhibition of pyrogallol autoxidation caused by SOD can be employed as a rapid and convenient method for the determination of the SOD activity (Hanzhen et al. 2006). The principle is that pyrogallol can rapidly autoxidize under an alkaline condition, releasing O_2_^−^ and generating a color-bearing intermediate product. The reaction starts with a yellow–green color and turns yellow after a while, with a linear time of 3–4 min. The addition of SOD inhibits the rate of autoxidation and the absorbance of the solution is measured at 325 nm. One unit of SOD activity is defined as the amount of enzyme required to inhibit the autoxidation of pyrogallol to 50%.

The bacterial solution was mixed with 50 mM Tris–EDTA at pH 8.2. The final OD_600_ of the solution was 2 in a 2.5 mL system. After preincubation for 5 min at 25 °C, the reaction was initiated by the addition of 10 μL of 50 mM pyrogallol solution in 10 mM HCl. The change of absorbance at 325 nm was measured every 1 min for 5 min at 25 °C, and the increase in absorbance per minute was calculated within the linear range (Liu et al. 2011). Tris–HCl buffer solution was prepared as a control. All measurements were made in triplicate.

To determine the cell surface displayed enzyme activity, we collated data from pyrogallol autoxidation. The Eqs. (1) and (2) were calculated as follows:

1 $${\text{Inhibition}}\,\left( \% \right) = \frac{{\frac{\Delta A_1 }{{\Delta t}} - \frac{\Delta A_2 }{{\Delta t}}}}{{\frac{\Delta A_1 }{{\Delta t}}}} \times 100\%$$

2 $${\text{SOD enzymatic activity}}\,\left( {\text{U/mg}} \right) = \frac{{{\text{Inhibition}}}}{50\% } \times \frac{V}{{V_0 V_{\text{S}} }}$$

where Δ*A*_1_/Δ*t* is the reaction rate of pyrogallol autoxidation; Δ*A*_2_/Δ*t* is the reaction rate of pyrogallol autoxidation with adding the sample. *V* is the total volume of the reaction solution; *V*_s_ is the volume of sample; *V*_0_ is the defined volume of viability unit (1 mL).

### Immunofluorescence observation

The display of E-tag short peptide on the outer membrane surface of *C. necator* was detected by fluorescence microscope (DM5000B 1030, Leica, Germany). Briefly, different recombinant strains were grown in LB medium and induced with 2.5 g/L arabinose at the OD_600_ of 1.5. The solution was aspirated for another 24 h of growth and then used for immunofluorescence experiments.

Bacterial cells were washed twice with phosphate buffered saline (PBS), fixed in 4% paraformaldehyde, and then sufficient washed again with PBS. After blocked with QuickBlock™ blocking buffer, the cells were incubated overnight at 4 °C with a rabbit anti-E-tagged polyclonal antibody, which connected with a goat anti-rabbit conjugated Alexa Fluor 488 and incubated for 2 h at the room temperature. Finally, the cells were washed and resuspended with 500 μL PBS for detection. The cells were also observed under a fluorescence microscope after immunofluorescence staining. Photographs were taken under both white light and green fluorescence filters.

### Autotrophic growth

A 100 mL glass serum bottle with a butyl rubber stopper was sterilized at 121 °C for 20 min and inoculated with 40 or 60 mL inoculum. The bottle was flushed with a gas stream of 70% H_2_, 10% O_2_, and 10% CO_2_. The strain was grown at 30 °C in an optimized medium solution that contained 6.74 g/L Na_2_HPO_4_·7H_2_O, 1.5 g/L KH_2_PO_4_, 1.0 g/L (NH_4_)_2_SO_4_, 80 mg/L MgSO_4_·7H_2_O, 1 mg/L CaSO_4_·2H_2_O, 0.56 mg/L NiSO_4_·7H_2_O, 0.4 mg/L ferric citrate, 200 mg/L NaHCO_3_, and 50 mg/L FeSO_4_ with the pH of 7.0–7.5. The mineral solution was sterilized with a sterile filter (0.22 μm) for later use. The sealed bottle was shaken at 600 rpm and 30 °C in a constant temperature water bath. Samples were extracted every 24 h to determine OD_600_. And the samples were centrifugally collected in preparation for the following tests.

### Electrochemical cell construction

The electrochemical tests were performed using a CHI 1000C potentiostat (CH Instruments Inc., Shanghai, China). The cell consisted of a 15 cm^2^ titanium electrode in a 130-mL anodic chamber containing 1 mM H_2_SO_4_ (pH 2.0), a 15 cm^2^ nickel electrode in a 130-mL cathodic chamber containing medium solution (pH 7.0–7.5), and a Nafion 212 membrane placed in between. The initial OD_600_ were controlled at 0.2–0.3. The final constant current was measured at 10–20 mA and a voltage of cathode was − 2.5 V (vs. Ag/AgCl). The cultivations were performed at 30 °C under stirring at 300 rpm while being sparged at 10 mL/min with a stream of gas containing 50% CO_2_ and 50% O_2_. The 0.43 g/L of SOD and 0.023 g/L of CAT were added during the first 2 days to ensure the survival of the strain. The 2.5 g/L of L-arabinose was added when OD_600_ reached to 1.5 to induce growth and stop enzyme supplementation. Samples were tested every 24 h to determine OD_600_ for a total of 7 days. All measurements were made in triplicate.

To evaluate stability, enzyme solutions (SOD and CAT) were incubated at 30 °C in BES for 7 days, and at fixed time intervals, sample aliquots were withdrawn for the activity test. The activity of SOD or CAT was determined by the change in pyrogallol or hydrogen peroxide at 325 or 240 nm.

### Comparison of the ability to scavenge ROS

The SOD and CAT activities were determined using the spectrophotometric method by determining of H_2_O_2_ with an enzyme-coupled assay using horseradish peroxidase (Martinez-Martinez et al. 2008). To measure the H_2_O_2_ content for each time period, 40 μL of electrolyte aqueous solution was mixed with 40 μL of SOD or CAT, and transferred to EP tubes containing 80 μL substrate solutions with the following final concentrations: 0.4 mM 4-aminoantipyrine, 7 mM phenol, 10 U/mL horseradish peroxidase and 500 mM PBS (pH 8.0). After 30 min of reaction, the absorbance of the solution was measured at 505 nm.

### PHB content analysis

*C. necator* was cultured in BES for 7 days, collected and centrifuged, washed with 0.9% NaCl solution and resuspended to a final OD_600_ of 10. After centrifugation of the heavy suspension, the precipitate was dried at 70 °C, followed by the addition H_2_SO_4_ and lysed at 95 °C for 30 min. The lysate was separated on the HPX-87H chromatographic column with a mobile phase of 5 mM H_2_SO_4_ at a flow rate of 0.6 mL/min (Zhang et al. 2023). The column temperature was 40 °C and the detection wavelength was 210 nm.

## Results and discussion

### Comparison of *C. necator* H16 and CMS

**Fig. 1:**
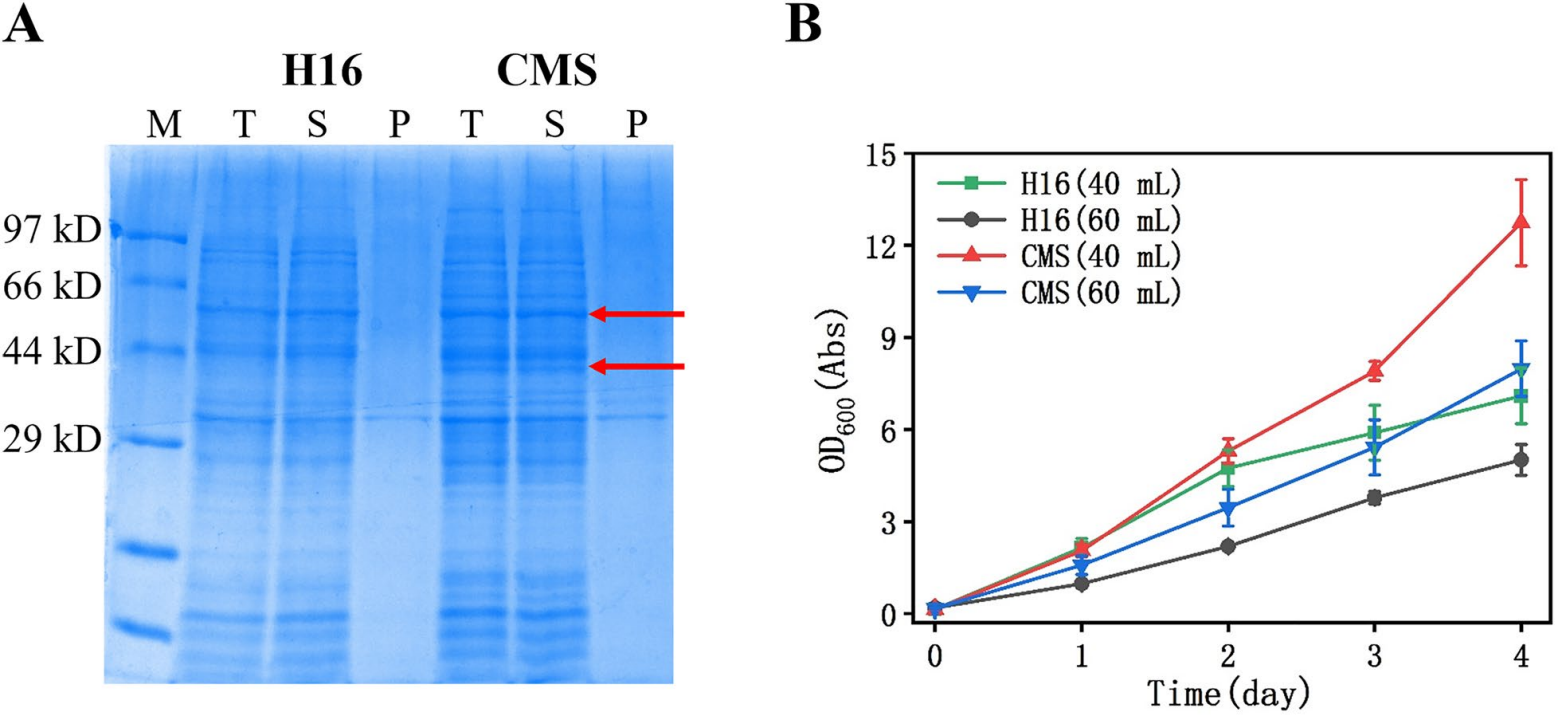
Comparison of *C. necator* H16 and CMS. **A** SDS-PAGE of proteins in the cell; M: marker; T: total protein; S: supernatant; P: pellet. **B** The growth of *C. necator* H16 and CMS (The volume indicates the fermentation broth volume)

When the organic carbon source in the environment is lacking, *C. necator* can grow rapidly under the conditions of CO_2_, H_2_ and O_2_. It is commendable that unlike many hydrogenases sensitive to oxygen, the hydrogenase derived from *C. necator* is resistant to oxygen. *C. necator* H16 produces four distinct [NiFe]-hydrogenases that each serves unique physiological roles (Burgdorf et al. 2005; Cramm 2009). For example, membrane-bound hydrogenase (MBH) is composed of HoxG (PHG002, 68.8 kD) and HoxK (PHG001, 39.5 kD) structural subunits, which are anchored to the membrane by HoxZ. Soluble hydrogenase (SH) consists of four subunits: HoxH (PHG091, 54.9 kD), HoxY (PHG090, 22.9 kD), HoxU (PHG089, 59.6 kD), and HoxF (PHG088, 66.7 kD), which deliver protons and electrons to NAD^+^ to synthesize NADH for cell growth and biosynthetic reactions. Compared with H16, CMS strengthens the promoter of MBH and SH gene clusters (Li et al. 2020). After expression in H16 and CMS, the homogeneity of these enzymes was examined by SDS-PAGE (Fig. 1a). Comparing to the control of H16, the CMS supernatant sample showed two significant bands marked with red arrows. This indicates that hydrogenase expression is greatly enhanced in CMS.

The metabolic features of autotrophic HOB allow them to grow on H_2_ and O_2_, while fixing CO_2_ into target products. For SCP accumulation, a completely stirred tank reactor or a sequencing batch reactor was used during the batch as well as continuous experiment (Matassa et al. 2016). Notably, the gas mixture of H_2_, CO_2_ and O_2_ was optimized with a ratio of 7:1:1 (Dou et al. 2019; Ruuskanen et al. 2021). The flow rate of the gas mixture was controlled at 0.5 VVM. The H_2_ was supplied by a generator, and the CO_2_ and O_2_ were provided from gas tanks. Since the result of filling 40 mL of liquid was better than that of filling 60 ml, we speculated that a sufficient head space for gas may be benefit for bacterial growth (Fig. 1b). The result also indicates that the growth of CMS is better than H16, which should be attributed to the improvement in the expression of MBH and SH of the former (Li et al. 2020).

### Selection of surface displayed enzymes

**Fig. 2:**
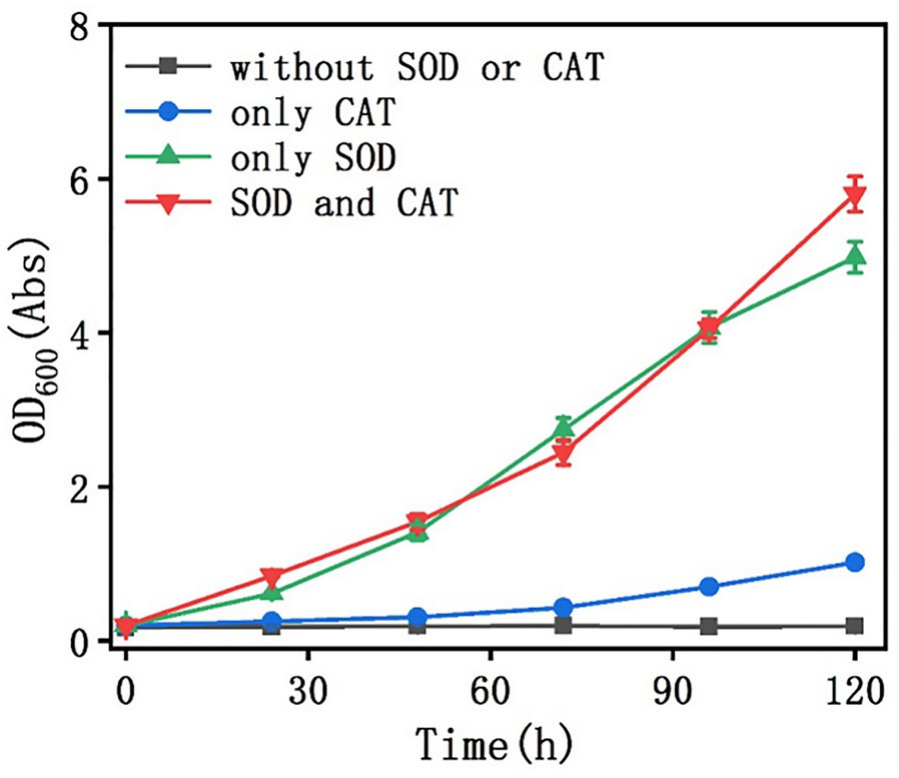
Effect of SOD or CAT on the cell growth in BES. *C. necator* growth curves in four different cathode chambers with adding SOD, CAT, SOD and CAT, or none, respectively

In BES, *C. necator* is difficult to survive due to the ROS generated during the electrolysis, which is harmful to cell growth (Additional file 1: Fig. S1). To mitigate this issue, protective antioxidant enzymes like SOD and CAT are often used (Torella et al. 2015). Herein, we investigated the effects of adding SOD and CAT to the cathodic chamber of a three-electrode system on the growth of *C. necator* CMS (Fig. 2). It clearly shows that the growth rate of the strain with only exogenous SOD or both SOD and CAT increased significantly compared to others. The impact of CAT, in contrast, appears to be very limited. These findings point to the conclusion that SOD protects cells from ROS damage much more effectively than CAT, at least in our case. Therefore, we assumed that cathodically generated O_2_^−^ was more cytotoxic in BES, suggesting the necessity to display SOD on the cell surface. As the control, the equivalent unit of free SOD was used to evaluate the advantage of cell surface display. We firstly performed the enzyme activity assay using free SOD to find the amount of enzymes that exhibited the same activity as that of the surface displayed enzymes. Then, we cultured the CMS strain in BES. When OD_600_ = 1.5, free SOD with the same enzyme activity as the surface displayed SOD was added every day to observe the growth status. It can be seen from Additional file 1: Fig. S2 that although the enzyme activity is similar, the effect of free SOD on CMS growth is much smaller than that of the surface displayed enzymes, presumably because the protective film formed by the surface displayed enzymes can effectively protect *C. necator* from the attack of O_2_^−^.

In addition, the stability of SOD and CAT was also evaluated. As shown in Additional file 1: Fig S3A, the CAT and SOD could retain 60–80% activity in BES after 24 h. Therefore, the SOD and CAT were added during the first 2 days to make the strain grow. Subsequently, H_2_O_2_ was measured using a spectrophotometric method and O_2_^−^ was determined via H_2_O_2_ measurement with an enzyme-coupled assay using horseradish peroxidase. From Additional file 1: Figs. S3B and S3C, it can be found that O_2_^−^ can be removed by adding SOD but then restored after a while, while H_2_O_2_ can be removed effectively by adding CAT. This result suggests that the O_2_^−^ is more difficult to be removed and display the SOD on the cell surface constantly could be a viable strategy.

### Influences of promoters, signal peptides and linker on surface displayed SOD

Consist with previous studies, autotransporters which have distinct functions in their native hosts play the most prominent role in the anchor proteins in many Gram-negative bacteria (Tozakidis et al. 2015). The functions of FhuA, OmpA, Lpp-OmpA and IgA on the cell surface display of *C. necator* were compared, and quantitative research demonstrated that IgA was a more effective anchor protein than others. (Tsai et al. 2015). Thus, IgA protease autotransporter was used in this study. To analyze the influence of the promoter and signal peptide on protein secretion by the autotransporter pathway, the *C. necator* CMS strains were transformed with plasmids BBa_J23100-SOD-E-tag-IgAβ, pBAD-pelB-SOD-E-tag-IgAβ, T7-SOD-E-tag-IgAβ and pBAD-pelB-SOD-E-tag-IgAβ.

**Fig. 3:**
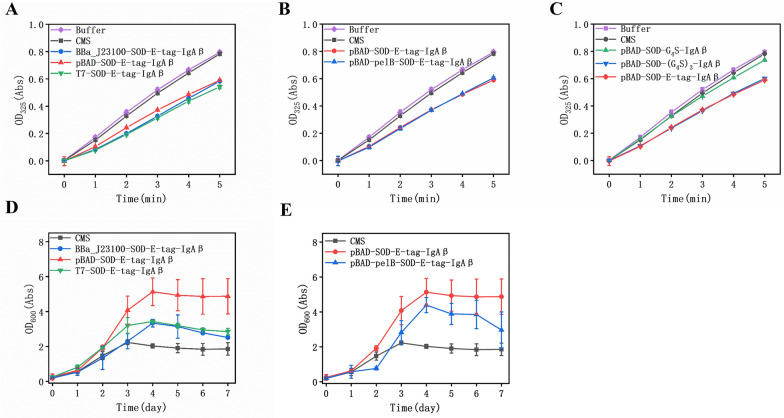
Comparison of the enzyme activity and growth displayed of the recombinant *C. necator* CMS strains. **A** Comparison of enzyme activities from the strains using different promoters; **B** Effect of the presence or absence of the signal peptide pelB on enzymatic activity; **C** Comparison of enzyme activities from the strains containing different linkers for the displayed SOD; **D** Comparison of strain growth using different promoters; **E** Effect of the presence or absence of the signal peptide pelB on growth

Initially, three promoters with different expression strengths were used to obtain the optimal one with the strongest displayed SOD activity. The result indicates that T7 expressed higher enzyme activity than BBa_J23100 or pBAD (Fig. 3a). After cultured three *C. necator* strains in BES, we found that the pBAD-driven strains showed better tolerance to O_2_^−^ and had a higher survival rate in terms of 7-day growth pattern (Fig. 3d). This result is slightly different from the activity assay (Fig. 3a), possibly due to a lower cell survival rate under the strong promoter. Similar result was also found in *E. coli* (Dong et al. 1995; Kurland and Dong 1996). These results suggest that the combination of IgAβ and pBAD promoter is the best choice for SOD displayed on *C. necator* CMS. To verify whether the expression of SOD induced by 2.5 g/L of arabinose was the optimal, we cultured CMS strain containing pBAD-SOD-E-tag-IgAβ in LB for 24 h and used different concentrations of arabinose. Additional file 1: Fig. S4 shows that strain growth is slightly better when 2.5 g/L arabinose is added for induction. Arabinose concentration had little effect on growth. This induction condition was also reported in another study (Beilharz et al. 2023). Next, we tested the effect of signal peptides on the strain growth. Previous study indicated that pelB was essential for the IgAβ-anchored system (Valls et al. 2000). However, our experiment result showed that enzymatic activity of displayed SOD is the same with or without the presence of pelB (Fig. 3b), and the strain containing pelB performs even worse than that without pelB (Fig. 3e). The reason for this result may be hyposecretion, which was confirmed by fluorescence analysis.

As an indispensable component of recombinant fusion proteins, linkers have been demonstrated to be of increasing importance in the construction of stable and bioactive fusion proteins (Chen et al. 2013). It is generally believed that the length of linker is 5–25 amino acid residues, which can provide enough space for two proteins, and therefore is beneficial to the folding and stability of proteins. An example of the most widely used flexible linker has the sequence of (Gly–Gly–Gly–Gly–Ser)_*n*_. In this experiment, the E-tag was used as both the immunofluorescence tag and the linker. To verify whether it had the function as a linker, we constructed a strain containing a flexible linker Gly_4_Ser or a triple-repeat of this linker (Gly_4_Ser)_3_, and compared it with the strain containing the E-tag. The results of enzyme activity show that the strain containing Gly_4_Ser has the weakest SOD activity, while (Gly_4_Ser)_3_ and the E-tag which has a similar number of amino acids exhibit almost the same enzyme activity (Fig. 3c).

Taken together, these results suggest that pBAD-SOD-E-tag-IgAβ is the best recombinant strain with an enzymatic activity of 21 U/mg. The growth rate of *C. necator* CMS displayed with SOD can achieve 4.9 ± 1.0 of OD_600_ by 7 days, equivalent to 1.7 ± 0.3 g/L DCW, and the production rate is 0.24 ± 0.04 g/L/d DCW, which shows around 2.7-fold increase than the original *C. necator* CMS (1.8 ± 0.3 of OD_600_).

### Fluorescence analysis

**Fig. 4:**
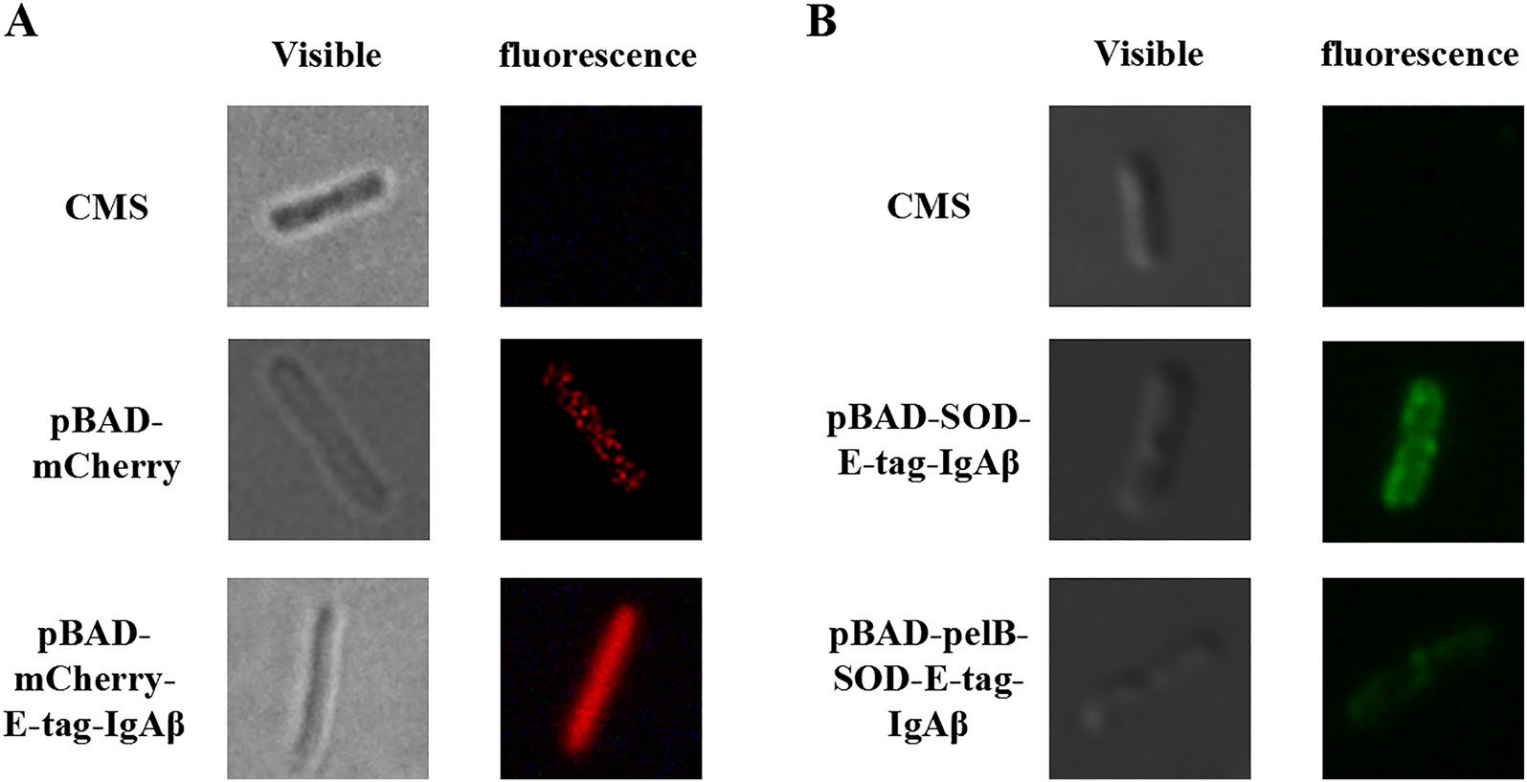
Fluorescence analysis.** A** Fluorescence micrographs of the CMS strains with surface displayed mCherry (bottom), intracellularly expressed mCherry (middle) and CMS only (top). Phase contrast image on left and fluorescence image on right; **B** Immunofluorescence microscopy images of the CMS strains expressing pBAD-SOD-E-tag-IgAβ and pBAD-pelB-SOD-E-tag-IgAβ

Microbial cell-surface display systems allow target proteins or peptides to be displayed on the surface of microbes through the fusion with anchor proteins. Because of its decreased background for intracellular imaging, the mCherry was chosen as a reporter to replace SOD for the fluorescence microscopy study. As shown in Fig. 4a, there is a fluorescent halo in fluorescence microscopy on *C. necator* CMS when mCherry is anchored to the outer membrane, compared to the strain with mCherry but without the anchor protein. This suggests that the protein is displayed uniformly across the cell surface. Instead, the expression of unanchored mCherry is distributed diffusely throughout the cell. This further suggests the important role of IgAβ as an anchor protein module.

To confirm whether the fusion proteins were properly translocated to the surface, the different CMS strains harboring SOD-E-tag plasmids were further analyzed by immunofluorescence (Fig. 4b). The primary antibody used was rabbit anti-E antibody and the secondary antibody was goat anti-rabbit IgG with Alexa Fluor 488 labeling, and the original CMS strain was used as a control. It was found that only the green fluorescence-labeled anchoring protein E-tag-IgAβ appeared on the cell surface on fluorescence images. These indicate that the anchor module, IgAβ, is displayed on the cell surface and the fusion protein is expressed in a non-degraded and active form. Moreover, the fluorescence intensity of the pelB-containing strain is noticeably lower, showing that pelB is inefficient in this case.

### Validation of surface displayed SOD

**Fig. 5:**
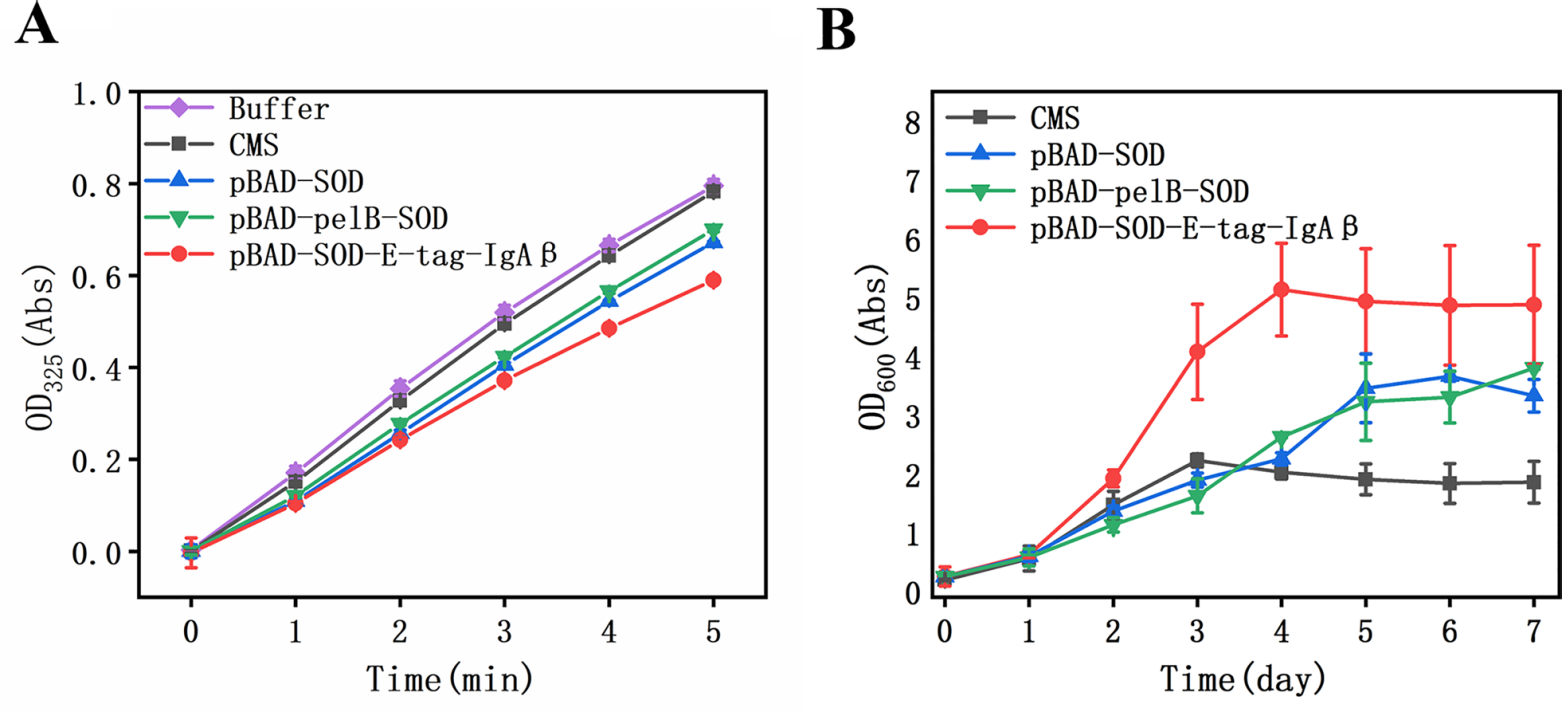
Comparison of enzyme activities and growth of three recombinant strains. **A** Comparison of SOD enzyme activities of three strains; **B** comparison of growth of three strains

We took advantage of the ability that SOD may convert O_2_^−^ to hydrogen peroxide as a way in tackling the issue of the existence of ROS in BES. For comparison, extracellular secretion of SOD and intracellular overexpression of SOD were also conducted using the strains containing the plasmids of pBAD-SOD and pBAD-pelB-SOD. Both enzyme activity assay (Fig. 5a) and the growth (Fig. 5b) results reveal that the strain with the surface displayed SOD outperforms significantly to the other two in BES. The effects of extracellular secretion or intracellular overexpression of SOD seem to be the same. The reason might be that extracellular secretion released into the solution or intracellular overexpression restrained in the cytoplasm of SOD are insignificant, which cannot prevent O_2_^−^ from breaking the outer membrane. In contrast, it is clear that SOD proteins are evenly displayed on the outer membrane of *C. necator* from the fluorescence experiments, which form a shield to protect the strain as expected.

Compared with using a porous ceramic cup (Li et al. 2012), this inexpensive biological shield can quench ROS to some extent before they reach the cells, resulting an enhanced cell growth. Cell viability can be saved by adding CAT (Torella et al. 2015). In addition, synthesis of anti-free radical substances such as lycopene can also improve *C. necator* growth in BES and the lycopene strain can reach ~ 2.0 of OD_600_ (Wu et al. 2022). However, in our study, the effect of using SOD is much better and the strain displayed with SOD can reach ~ 4.9 of OD_600_ at the optimal condition (Fig. 5b). Moreover, the use of surface display technology avoids the problem of poor enzyme stability and the need of enzyme supplementation.

To check whether the overexpression of SOD exert negative effects on cell metabolism, we investigated the growth and the PHB production level of the strain. As shown in Additional file 1: Fig. S5, the growth state of the overexpression strain cultured for 24 h after the induction is not as good as those of the surface displayed and original CMS strains. The PHB content of original CMS strain is found to be similar to that of surface displayed strain, but much higher than that of overexpressed strain. It suggests that the enzyme overexpression may affect the formation of intracellular metabolites.

## Conclusions

In this study, we investigated the culture of *C. necator* in BES. Specifically, we designed a number of *C. necator* strains displayed with SOD enzymes and investigated the impacts of promoters, signal peptides and linkers to solve the issue of ROS generated in BES. Moreover, it was demonstrated that cell surface display outperformed overexpression or secretory expression of the enzyme. The results show that the growth rate of *C. necator* CMS with pBAD-SOD-E-tag-IgAβ can achieve 4.9 ± 1.0 of OD_600_ by 7 days, equivalent to 1.7 ± 0.3 g/L DCW, and the production rate is 0.24 ± 0.04 g/L/d DCW, around 2.7-fold increase than the original *C. necator* CMS (1.8 ± 0.3 of OD_600_). This study can provide a novel solution for the growth of strains and the production of chemicals in BES. In future studies, it is needed to optimize the cell surface display system of *C. neactor*, and study the mechanism of ROS inhibiting the growth of *C. neactor*, so as to further improve the growth of strains in BES.

### Supplementary Information

**Additional file 1:** **Fig. S1** C. necator growth in BES at -2.5 V (vs. Ag/AgCl) without the addition of SOD or CAT. **Fig. S2** Influence of free SOD on CMS growth in BES. Free SOD with the same enzyme activity as the displayed SOD was added to observe the growth of CMS in BES. **Fig. S3** Stability of SOD or CAT and ability to scavenge O_2_.-or H_2_O_2_. (A) Stability of SOD or CAT in BES; (B) Changes in the content of O_2_.- in BES after the addition of SOD; (C) Change of H_2_O_2_ content in BES after the addition of CAT. **Fig. S4** Influence of arabinose concentration on C. necator growth. (A) Influence of different concentrations of arabinose on the growth of C. necator; (B) Influence of arabinose (2.5 g/L) on CMS growth. **Fig. S5** Comparison of growth ability and PHB production of different strains. (A) Influence on the growth of different strains. The strains grown in LB for 24 h were collected to determine OD600. (B) Determination of PHB by HPLC. The strains grown in BES for 7 days were collected and subjected to HPLC after acid hydrolysis, and the peak absorption of PHB was observed and the relevant PHB content was obtained.

## Data Availability

The datasets used and/or analyzed during the current study are available from the corresponding author on reasonable request.

## Abbreviations

BES: Bioelectrochemical system
SCP: Single cell protein
HOB: Hydrogen-oxidizing bacteria
ROS: Reactive oxygen species
DCW: Dry cell weight
CAT: Catalase
SOD: Superoxide dismutase
O_2_^−^: Superoxide anion
SDS: Sodium dodecyl sulfate polyacrylamide gel electrophoresis
PBS: Phosphate buffered saline
