# Supplementary material for: Construction of Cupriavidus necator displayed with superoxide dismutases for enhanced growth in bioelectrochemical systems
Source: Bioresour Bioprocess. 2023 Jun 28;10(1):36. doi: 10.1186/s40643-023-00655-2 (PMC10992759; doi:10.1186/s40643-023-00655-2)
Supplement: Supplementary file 1 — Additional file 1: Fig. S1 C. necator growth in BES at -2.5 V (vs. Ag/AgCl) without the addition of SOD or CAT. Fig. S2 Influence of free SOD on CMS growth in BES. Free SOD with the same enzyme activity as the displayed SOD was added to observe the growth of CMS in BES. Fig. S3 Stability of SOD or CAT and ability to scavenge O2.-or H2O2. (A) Stability of SOD or CAT in BES; (B) Changes in the content of O2.- in BES after the addition of SOD; (C) Change of H2O2 content in BES after the addition of CAT. Fig. S4 Influence of arabinose concentration on C. necator growth. (A) Influence of different concentrations of arabinose on the growth of C. necator; (B) Influence of arabinose (2.5 g/L) on CMS growth. Fig. S5 Comparison of growth ability and PHB production of different strains. (A) Influence on the growth of different strains. The strains grown in LB for 24 h were collected to determine OD600. (B) Determination of PHB by HPLC. The strains grown in BES for 7 days were collected and subjected to HPLC after acid hydrolysis, and the peak absorption of PHB was observed and the relevant PHB content was obtained. [file 40643_2023_655_MOESM1_ESM.docx]

**Supporting Information**

**Construction of *Cupriavidus necator* displayed with superoxide dismutases for enhanced growth in bioelectrochemical systems**

Ke Chen^a,d#^, Chunling Ma^c,d,e#^, Xiaolei Cheng^b,d#^, Yuhua Wang ^b,d^, Kun Guo^e^, Ranran Wu^b,d^, Zhiguang Zhu^b,d^*

^a^ Key Laboratory of Industrial Fermentation Microbiology, Ministry of Education, Tianjin Key Laboratory of Industrial Microbiology, The College of Biotechnology, Tianjin University of Science and Technology, Tianjin 300457, China

^b^ University of Chinese Academy of Sciences, 19A Yuquan Road, Shijingshan District, Beijing 100049, China

^c^ Haihe Laboratory of Synthetic Biology, 21 Xishiwudao, Tianjin Airport Economic Park, Tianjin 300308, China

^d^ Key Laboratory of Engineering Biology for Low-Carbon Manufacturing, Tianjin Institute of Industrial Biotechnology, Chinese Academy of Sciences, 32 Xiqidao, Tianjin Airport Economic Park, Tianjin 300308, China

^e^ School of Chemical Engineering and Technology, Xi'an Jiaotong University, Xi'an 710049, China

^#^ These authors contribute equally to this work.

* Corresponding Author: Zhiguang Zhu (zhu_zg@tib.cas.cn),

ORCID: 0000-0002-6625-5087

Tel: (+86)-022- 2482 8797; Fax: (+86)-022-8486 1926


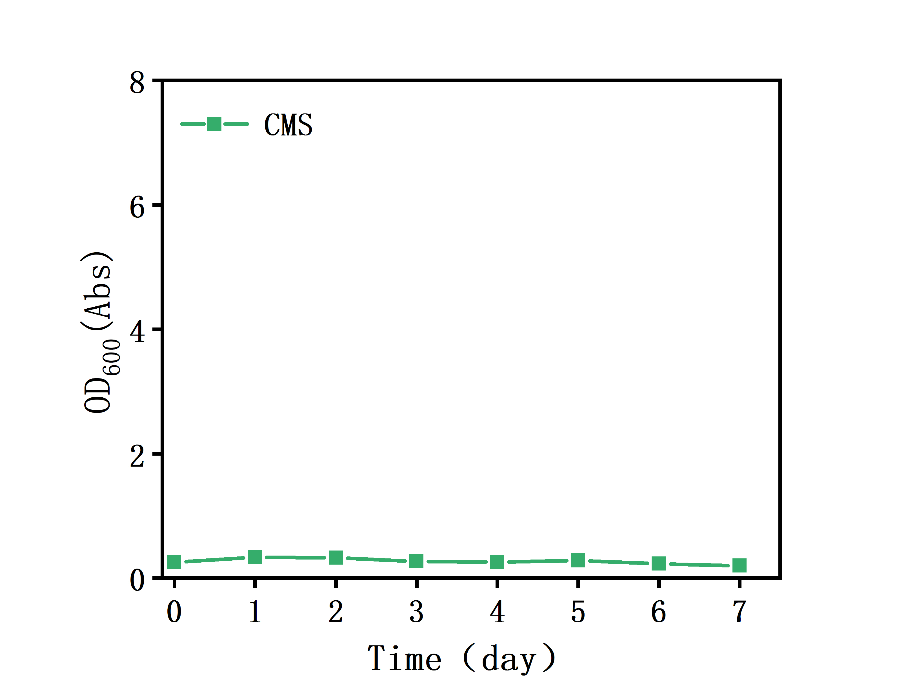


**Fig. S1** *C. necator* growth in BES at -2.5 V (vs. Ag/AgCl) without the addition of SOD or CAT.


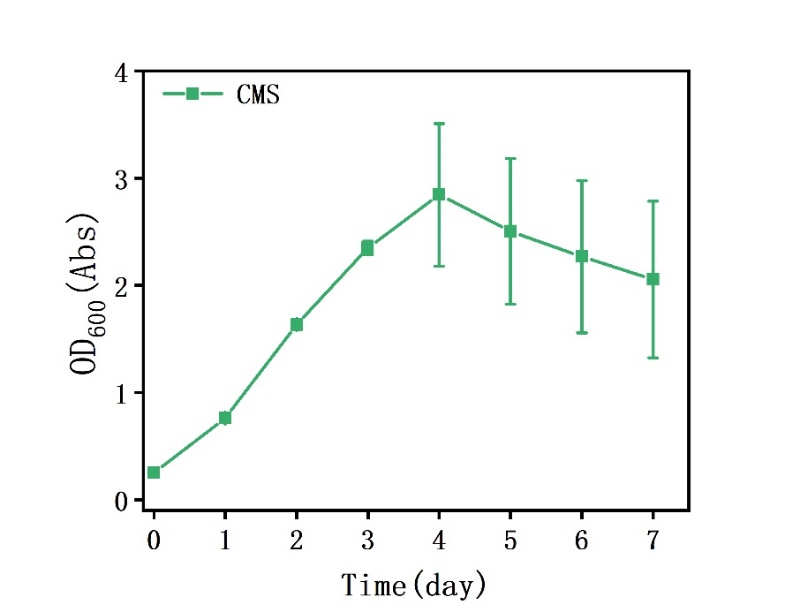


**Fig. S2** Influence of free SOD on CMS growth in BES. Free SOD with the same enzyme activity as the displayed SOD was added to observe the growth of CMS in BES.


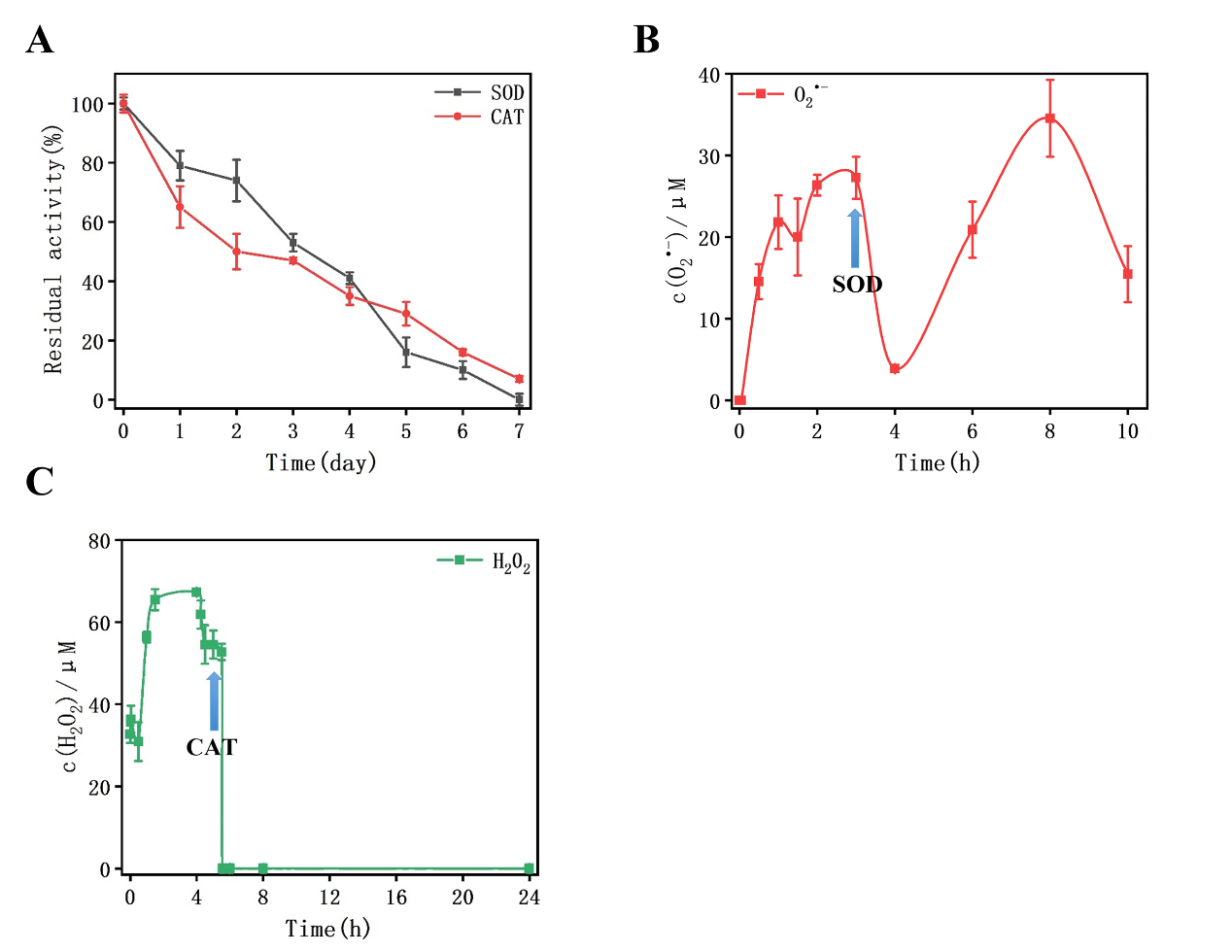


**Fig. S3** Stability of SOD or CAT and ability to scavenge O_2_^.-^or H_2_O_2_. (A) Stability of SOD or CAT in BES; (B) Changes in the content of O_2_^.-^ in BES after the addition of SOD; (C) Change of H_2_O_2_ content in BES after the addition of CAT.


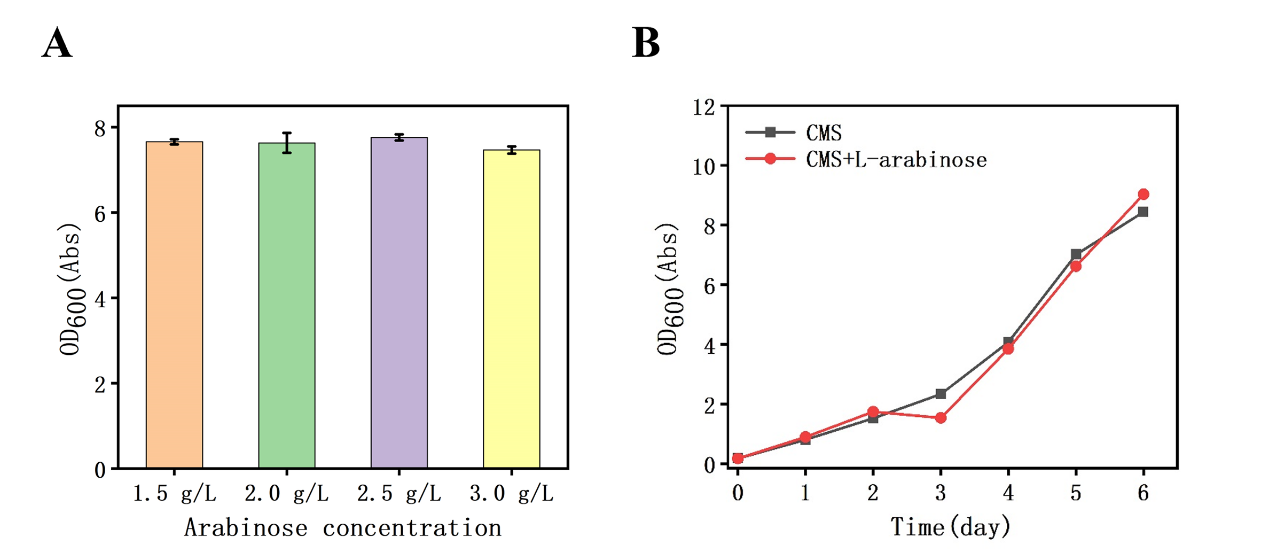


**Fig. S4** Influence of arabinose concentration on *C. necator* growth. (A) Influence of different concentrations of arabinose on the growth of *C. necator*; (B) Influence of arabinose (2.5 g/L) on CMS growth.


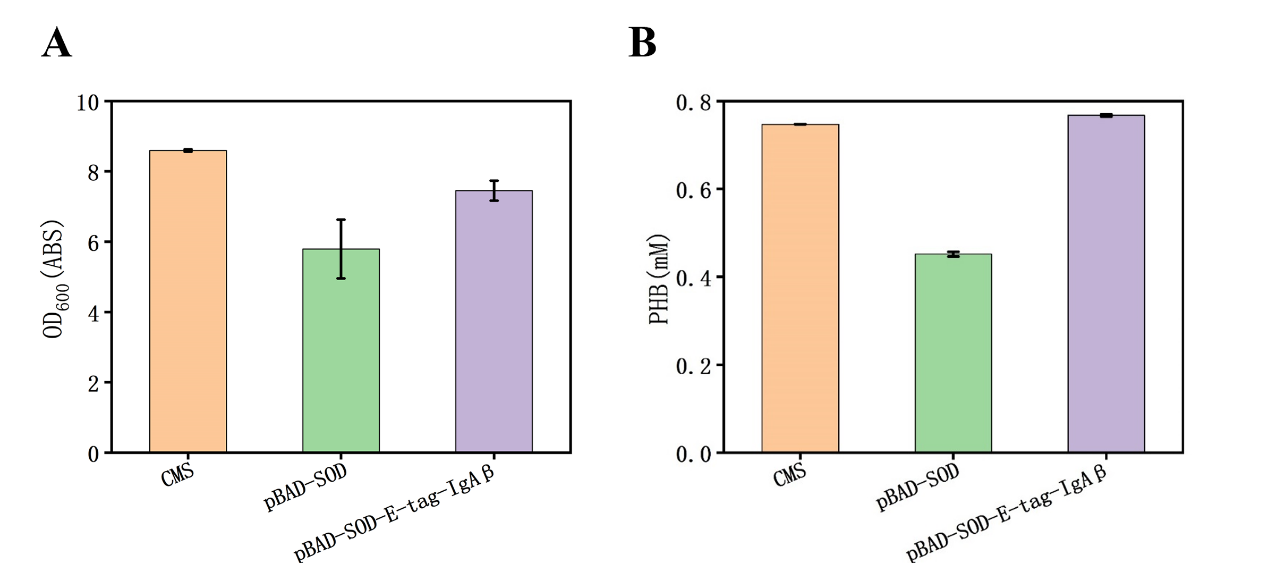


**Fig. S5** Comparison of growth ability and PHB production of different strains. (A) Influence on the growth of different strains. The strains grown in LB for 24 h were collected to determine OD_600_. (B) Determination of PHB by HPLC. The strains grown in BES for 7 days were collected and subjected to HPLC after acid hydrolysis, and the peak absorption of PHB was observed and the relevant PHB content was obtained.

**Tables**

**Table. S1** Primers used in this study

| Primers | Sequence (5’→3’) |
| --- | --- |
| BBa_J23100-SOD-E-tag-IgAβ construction | |
| IF-SOD | CTAGCTTTAAGAAGGAGATATACATATGCCGTACCCGTTCAAGCTTCCTG |
| IR-SOD-E-tag-IgAβ | GATATGGTACTGGTGCTCCCATATGGGCCTTCTTGAAGAACTCCTCGGCC |
| VF-SOD-E-tag-IgAβ | GGCCGAGGAGTTCTTCAAGAAGGCCCATATGGGAGCACCAGTACCATATC |
| VR-SOD | CAGGAAGCTTGAACGGGTACGGCATATGTATATCTCCTTCTTAAAGCTAG |
| VF-E-tag-IgAβ | AATACAGATTCGTTTCTAACTCGAGTTCATCGCCGACCGACTGCCCTTAT |
| IR-E-tag-IgAβ | ATAAGGGCAGTCGGTCGGCGATGAACTCGAGTTAGAAACGAATCTGTATT |
| pBAD-SOD-E-tag-IgAβ construction | |
| IF-pBAD | AAGCATTCTGTAACAAAGCGGGACCAAAGCCATGACAAAAACGCGTAACA |
| IR-pBAD | ATTCCTAGGGCCCAAAAAAACGGGTATGGAGAAACAGTAGAGAGTTGCGA |
| VF-pBAD | TCGCAACTCTCTACTGTTTCTCCATACCCGTTTTTTTGGGCCCTAGGAAT |
| VR-pBAD | TGTTACGCGTTTTTGTCATGGCTTTGGTCCCGCTTTGTTACAGAATGCTT |
| T7-SOD-E-tag-IgAβ construction | |
| IF-T7-SOD-E-tag-IgAβ | TTCTGTAACAAAGCGGGACCTAATACGACTCACTATAGGGGAATTGTGAGCGGATAACAATTCCACCCGTTTTTTTGGGCCCTA |
| IR-T7-SOD-E-tag-IgAβ | TAGGGCCCAAAAAAACGGGTGGAATTGTTATCCGCTCACAATTCCCCTATAGTGAGTCGTATTAGGTCCCGCTTTGTTACAGAA |
| pBAD-pelB-SOD-E-tag-IgAβ construction | |
| IF-Kpn1-pBAD-pelB | CGGGGTACCATGAAATACCTGCTGCCGACCGCTGCTGCTGGTCTGCTGCTCCTCGCTGCCCAGCCGGCGATGGCCATGCCGTACCCGTTC |
| IR-Kpn1-pBAD-pelB | CGGGGTACCCATATGTATATCTCCTTCTTAAAGT |
| pBAD-mCherry-E-tag-IgAβ construction | |
| IF-pBAD-mCherry-E-tag-IgA | ACTTTAAGAAGGAGATATACATATGAGCAAGGGCGAGGAGGATAACATGG |
| IR-pBAD-mCherry-E-tag-IgA | GATATGGTACTGGTGCTCCCATATGCTTGTACAGCTCGTCCATGCCGCCG |
| VF-pBAD-mCherry-E-tag-IgA | CGGCGGCATGGACGAGCTGTACAAGCATATGGGAGCACCAGTACCATATC |
| VR-pBAD-mCherry-E-tag-IgA | CCATGTTATCCTCCTCGCCCTTGCTCATATGTATATCTCCTTCTTAAAGT |
| pBAD-mCherry construction | |
| IF-pBAD-mCherry | ACCGGCGGCATGGACGAGCTGTACAAGTAACTCGAGCAAATAAAACGAAAGGCTCAGTCG |
| IR-pBAD-mCherry | CGACTGAGCCTTTCGTTTTATTTGCTCGAGTTACTTGTACAGCTCGTCCATGCCGCCGGT |
| pBAD-SOD construction | |
| IF-pBAD-SOD | GAATAATTTTGTTTAACTTTAAGAAGGAGATATACATATGATGCCGTACCCGTTCAAGCTTCCTGACCTAGGCTACCCCT |
| IR-pBAD-SOD | AGGGGTAGCCTAGGTCAGGAAGCTTGAACGGGTACGGCATCATATGTATATCTCCTTCTTAAAGTTAAACAAAATTATTC |
| pBAD-pelB-SOD construction | |
| IF-pBAD-pelB-SOD | TGGCCGAGGAGTTCTTCAAGAAGGCCTAACTCGAGCAAATAAAACGAAAG |
| IR-pBAD-pelB-SOD | CTTTCGTTTTATTTGCTCGAGTTAGGCCTTCTTGAAGAACTCCTCGGCCA |
| pBAD-SOD- G_4_S-IgAβ construction | |
| IF-PSLI-G_4_S | GGAGGAGGAGGAAGCGGAGCACCAGTACCATATCCAGACC |
| IR-PSLI-G_4_S | GCTTCCTCCTCCTCCGGCCTTCTTGAAGAACTCCTCGGCC |
| pBAD-SOD-(G_4_S)_3_-IgAβ construction | |
| IF-PSLI-(G_4_S)_3_ | GGAGGAGGAGGAAGCGGAGGAGGAGGAAGCGGAGGAGGAGGAAGCGGAGCACCAGTACCATATCCAGACC |
| IR-PSLI-(G_4_S)_3_ | GCTTCCTCCTCCTCCGCTTCCTCCTCCTCCGCTTCCTCCTCCTCCGGCCTTCTTGAAGAACTCCTCGGCC |
